# Hernie para duodénale gauche: une cause rare d’occlusion intestinale aiguë

**DOI:** 10.11604/pamj.2020.36.326.24958

**Published:** 2020-08-24

**Authors:** Abdelkader Mizouni, Fathia Harrabi, Waad Farhat, Linda Ghabri, Mohamed Ben Mabrouk, Ali Ben Ali

**Affiliations:** 1 Service de Chirurgie Générale, CHU Sahloul de Sousse, Faculté de Médecine de Sousse, Université de Sousse, Sousse, Tunisie

**Keywords:** Hernie para duodénale, hernie interne, chirurgie générale, Paraduodenal hernia, internal hernia, general surgery

## Abstract

La hernie para duodénale gauche est une hernie interne congénitale qui se complique rarement par une occlusion intestinale aiguë et qui peut mettre en jeux le pronostic vital par ischémie des anses. Nous rapportons un cas d’un jeune homme de 28 ans pris en charge pour un syndrome occlusif. Le scanner abdominal a objectivé une occlusion intestinale aiguë haute, l’exploration peropératoire a trouvé des anses jéjunales incarcérées dans une hernie para duodénale gauche. Le collet a été fermé et les suites opératoires étaient simples. En conclusion, la hernie para duodénale gauche, est souvent diagnostiquée lors d’une complication, son traitement est chirurgical et préférentiellement par laparoscopie.

## Abstract (en)

Left paraduodenal hernia is a congenital internal hernia rarely complicated by acute intestinal occlusion and resulting, sometimes, in life-threatening loops ischemia. We report the case of a 28-year old man treated for occlusive syndrome. Abdominal computerized tomography (CT) scan objectified acute upper intestinal obstruction; intraoperative exploration found jejunal loops incarcerated into left paraduodenal hernia. The neck was closed and the operative suites were simple. Left paraduodenal hernia is often diagnosed when patients have complications. Surgery is the treatment of choice, in particular laparoscopy.

## Introduction

La hernie para duodénale gauche est une hernie interne congénitale [1, 2]. Elle représente les deux tiers des hernies para duodénales, qui elles-mêmes représentent la moitié des hernies internes. Souvent elle est asymptomatique mais rarement se complique d’occlusion intestinale aiguë [3]. La connaissance de cette anomalie par les chirurgiens peut réduire leurs morbi-mortalité. D’où l’intérêt de présenter ce cas rare.

## Patient et observation

**Figure 1:**
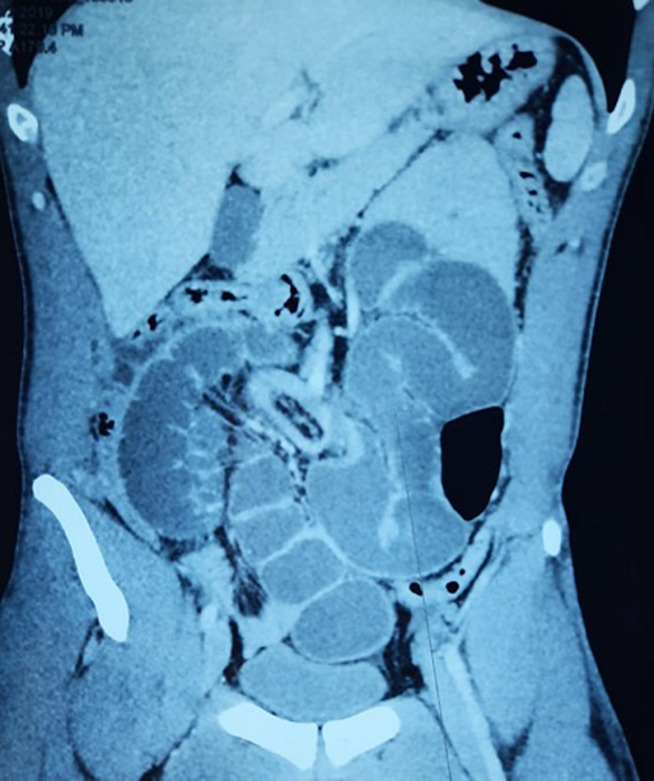
image d’une coupe scannographique sagittale

**Figure 2:**
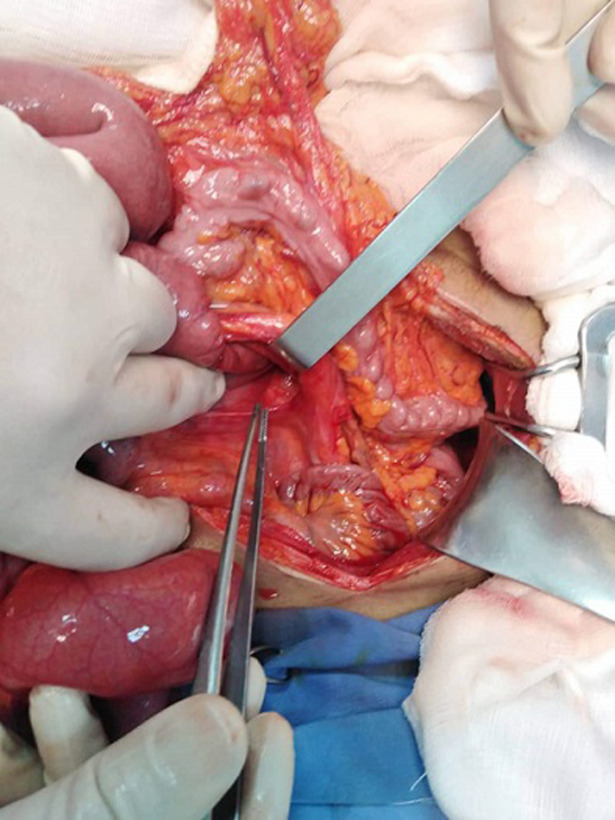
hernie para duodénale gauche (fossette de Landzert)

Un jeune homme de 28 ans, aux antécédents d’appendicectomie et de deux épisodes d’occlusion intestinale aiguë qui ont cédés après traitement médicale, a consulté les urgences pour un syndrome occlusif évoluant depuis un jour. A l’examen on a constaté des douleurs à la palpation de la région péri ombilicale avec une cicatrice de Mac Burney solide. L’examen biologique a objectivé un syndrome inflammatoire. Le scanner abdominal a confirmé le diagnostic d’une occlusion intestinale haute avec une distension modérée des anses grêliques iléales proximales en amont de deux niveaux transitionnels en bec dans la fosse iliaque gauche autour d’un signe de tourbillon médian sans signes de souffrance intestinale associée (Figure 1). Devant ce tableau le patient a été opéré en urgence par laparotomie, l’exploration peropératoire a objectivé une incarcération des anses jéjunales dans une hernie para duodénale gauche (Figure 2), le grêle a été libéré et les anses étaient de bonne vitalité. Le collet a été fermé par des points séparés par un fil résorbable. Les suites opératoires étaient simples.

## Discussion

La hernie para duodénale gauche résulte d’un défaut d’accolement des feuillets péritonéaux du fascia du treitz [2], dû à une anomalie de rotation de l’anse intestinale primitive pendant la phase embryonnaire qui entraine un défaut d’accolement entre le mésocolon gauche et le retro péritoine, ce qui aménage une fossette para duodénale gauche appelée la fosse de Landzert [2, 4]. La fosse est située à gauche du quatrième duodénum et postérieure par rapport à la veine mésentérique inférieure [4, 5]. Les hernies para duodénaux représentent 53% des hernies internes et la hernie para duodénale gauche représente 75% des hernies para duodénaux [3]. Souvent asymptomatique ou se manifeste par des douleurs abdominales vagues avec des troubles dyspeptiques [4, 6, 7]. Elles se compliquent rarement (0,2-0,9%) par l’incarcération des anses grêles dans la fossette de Landzert entrainant un syndrome occlusif par obstruction intestinale [3, 4]. Le diagnostic se fait par le scanner abdominal qui objective une agglutination des anses grêles à gauche de l’angle duodéno jéjunale de Treitz [1, 8]. Le traitement est une urgence chirurgicale [1, 3]. Il consiste à libérer la grêle incarcérée, évaluer la vitalité des anses et fermer le collet par points séparés au fil résorbable ou non [4, 6]. L’abord chirurgical peut être par voie ouverte [6] ou par laparoscopie qui offre moins de douleur postopératoire, un rétablissement précoce et un court séjour hospitalier [3].

## Conclusion

La hernie para duodénale gauche est une hernie interne congénitale rare, la plus fréquente des hernies para duodénaux, elle se complique rarement par une occlusion intestinale aiguë. Le diagnostic est scannographique. Son traitement est chirurgical préférentiellement par abord laparoscopique.

## References

[1] Falk GA, Yurcisin BJ, Sell HS (2010). Left paraduodenal hernia: case report and review of the literature. BMJ Case Rep.

[2] Peltier J, Page C, Havet E, LE Gars D, Mertl P, Foulon P (2004). Les fossettes paraduodénales?. étude anatomique et applications cliniques Morphologie.

[3] Schizas D, Apostolou K, Krivan S, Kanavidis P, Katsaros I, Vailas M (2019). Paraduodenal hernias: a systematic review of the literature. Hernia.

[4] Wakabayashi M, Kono S, Takahashi T (2018). Laparoscopic repair of acute small bowel obstruction due to left paraduodenal hernia: A case report. Int J Surg Case Rep.

[5] Martins A, Gonçalves Á, Almeida T, Gomes R, Lomba J, Midões A (2018). Left Paraduodenal Hernia. J Gastrointest Surg.

[6] Kotobi H, Echaieb A, Gallot D (2005). Traitement chirurgical des hernies rares. EMC-Chirurgie.

[7] Jin C, Mo J, Wang G, Jiang H, Feng Y, Wang S (2018). Paraduodenal hernia complicated with intussusception: case report. BMC Surg.

[8] Kulendran K, Keogh C, Chiam H-C (2019). Laparoscopic repair of a left paraduodenal hernia. ANZ J Surg.

